# Hand Microbial Flora of Hospitalized Children at the Beginning of Hospitalization and Before Discharge: A Cross-Sectional Study

**DOI:** 10.4314/ejhs.v30i6.4

**Published:** 2020-11

**Authors:** Emriye Hilal Yayan, Pınar Demırel Öner, Didem Coşkun Şımşek, Mürşide Zengın

**Affiliations:** 1 Department of Child Health and Diseases Nursing, Nursing Faculty, İnönü University, Malatya, Turkey. orcid.org/0000-0003-0075-4171; 2 Elazığ Fethi Sekin City Hospital, Elazığ, Turkey orcid.org/0000-0001-9592-5986; 3 Department of Child Health and Diseases Nursing, Health Sciences Faculty, Fırat University, Elazığ, Turkey orcid.org/0000-0003-0364-5667; 4 Department of Child Health and Diseases Nursing, Health Sciences Faculty, Adıyaman University, Adıyaman, Turkey. https://orcid.org/0000-0003-1453-6028

**Keywords:** Child hospitalized, hand hygiene, healthcare associated infection

## Abstract

**Background:**

Hospital infections in pediatric units increase the length of hospital stay and the use of antibiotics, and this causes exposure to more procedures. This study was aimed to determine the microorganisms represented in the hand flora of pediatric patients at the beginning of hospitalization and before discharge.

**Methods:**

The study was designed as a prospective cross-sectional study. This prospective study was performed with 124 pediatric patients. After completion of the admission procedures, an initial sample was taken from the hands of the hospitalized patients. Another sample was taken from the patients just before discharging.

**Results:**

Growth of coagulase-negative staphylococcus (CNS) was observed in the culture samples of 28 patients. Cultures from 23 patients showed different microorganisms such as Staphylococcus aureus, Escherichia coli, and S. epidermis. Examination of final discharge cultures showed CNS in 43 patients, S. aureus in 5 patients, E. coli in 8 patients, Acinetobacter baumannii in 11 patients, and Kocuria rhizophila, K. kristinae, Candida spp., Pseudomonas spp., and Enterococcus in 1 patient.

**Conclusion:**

The cultures from samples obtained at discharge showed the presence of antibiotic-resistant pathogenic microorganisms causing healthcare associated infection.

## Introduction

The World Health Organization reported that hundreds of millions of patients are affected by healthcare associated infections worldwide (1,2). Maintaining hand hygiene is a simple and effective method to prevent healthcare associated infections, which cause cross-infection and development of resistant pathogens. Ensuring optimum hand hygiene involves minimum costs (1).

Although all healthcare professionals are aware of healthcare associated infections, effective measures to prevent these infections are not successfully implemented in hospitals (3,4). Despite recommendations from the World Health Organization, hand hygiene compliance remains at 40% (4).

Hospital infections are caused by various microorganisms, including effective and longliving microorganisms (*Clostridium difficile*, *Acinetobacter baumannii, Staphylococcus aureus*, *methicillin-resistant S. aureus* (MRSA), *vancomycin-resistant enterococci* (VRE). Hospitals ensure cleaning of surfaces to prevent the spread of microorganism. Hospitals use different methods to clean the floor and disinfect the environment. The surfaces that support the reproductive of the environmental pathogens and normal flora are mechanically cleaned. However, these surfaces are at a high risk of rapid recontamination during routine practice (5).

Hospital infections in pediatric units increase the length of hospital stay and the use of antibiotics, and this causes exposure to more procedures (6,7). Pediatric patients are in contact with almost all healthcare professionals, especially physicians and nurses who are involved in their care. Children are at greater risk for infections. The rate of hospital infections is 6%–7% in pediatric patients and 4% in adults (8). The rates of hospital infection in pediatric patients vary in different countries, and they range from 6% to 29.6% (9–11). A study conducted in pediatric units in Europe showed that the incidence of nosocomial infection was 2.5%, ranging from 1% in general pediatric units to 23.6% in pediatric intensive care units (12). A multicenter study conducted in different pediatric hospitals in Switzerland showed that the rate of hospital infection was 6.7% (13). The rate of hospital infections in pediatric patients detected after discharge is 18% (14).

Studies conducted in daycare units reported that gastrointestinal and respiratory infections in children decreased after they were given the hand hygiene training (15–17). Despite the lack of microbiological findings, since children contract the same disease at the same time, this fact was associated with hand hygiene. However, since there is no research about hand hygiene in pediatric units and since these children are not followed up regularly after discharge, enough information about this case is not available. This study, therefore, aimed to determine the microorganisms represented in the hand flora of pediatric patients at the beginning of hospitalization and before discharge.

## Methods

**Study design and setup**: This prospective study was performed with 124 pediatric patients. The population of the study has consisted of the children 3–6 years old who were hospitalized in the pediatric unit of a university hospital between January and March 2017. The unit has consist of children with inpatient treatment. The unit consists of a 24-bed capacity with an intervention room, treatment room, doctor room and nurse room. The patients' rooms in the unit are designed to stay at least two children. Each patient room has two patient beds, two companion chairs, a toilet and a sink. While interventional procedures such as catheterization are performed in the intervention room, treatment and care applications are carried out in patient rooms. Children with respiratory, gastrointestinal, urogenital and neurologic system diseases stay in the unit. The average rate of admission to the clinic is 80%. Inclusion criteria of the study are children who interacted with other pediatric patients in the unit, from 3–6-year-old, and had no intellectual disability. Children admitted to the hospital for treatment, who meet the inclusion criteria, and whose written consent from their parents have been included in the study. The sample size of the study was calculated as 110 with 0.5 effect size and 0.05 error and the 0.95 representation power.

One hundred and thirty children who were hospitalized in the pediatric unit and who met the inclusion criteria of the study were included in the study. The study was conducted with 124 pediatric patients, because 4 of them were discharged from the hospital before 2 days and the parents of 2 children did not consent to participation in the study.

The hospital where the study was conducted had protocols for the prevention of hospital infection as determined by the Infection Board of the hospital. Healthcare professionals provide training for ensuring general and hand hygiene to all hospitalized children and their parents. In the context of hand hygiene training, it was stated that hands, including palms, fingers, and the full hand surface, should be washed with liquid soap and water for a minimum of 15 seconds. There are alcohol-based hand disinfectants in patients' rooms. Hand disinfectants were mounted 120 cm above the ground due to the risky behavior of children and for effective use by mothers.

**Measurements**: Two samples for bacteriological culture (initial culture and discharge culture) were taken from hands. After completion of the hospitalization procedures of the patients who visited our unit, an initial sample was taken from their hands. Subsequently, a final sample was taken just before discharging from pediatric patients. This was termed as discharge culture. The samples were compared to assess which bacteria had colonized the hands of the participants during the hospitalization and which corresponded to resident microbial flora.

Samples were collected from right hands (actively used by children) using sterile swabs pre-moistened with sterile serum physiologic solution. Samples were collected from areas that were more likely to be contaminated, such as inter-digital spaces and spaces under the nails and from the dorsum and palm of the hand. Data collection for the study from 124 pediatric patients took 66 days.

**Quality control and quality assurance**: The samples were studied in an accredited laboratory, and quality control criteria were met.

Swab samples were seeded on bloody agar and Eosin Methylene Blue (EMB) agar media. Colony counts, colony morphology, gram staining, catalase test, coagulase test, and biochemical characteristics were examined after incubation at 37ºC for 24–48 hours incubation. Antibiotic susceptibilities of these colonies were determined using the disk reduction method, if necessary.

**Statistical analysis**: The data analysis was performed using SPSS 18.0. Percentage distribution and arithmetic mean analyses were used for statistical analysis. The results were evaluated at a 95% confidence interval and at a significance level of p < 0.05.

**Ethical consideration**: All approvals were obtained from the institution where the study was conducted before commencing the study. Oral and written consents of parents of the children participating in the study were obtained. The ethics permission of this study was obtained from the Ethics Committee of İnönü University (2016/3-7).

## Results

The mean age of the pediatric patients participating the study was 5.42 ± 1.78 years. Table 1 presents some features of the pediatric patients. In the study, both of the average stay of children in the clinic and time taken to take one swab sample from a child were 3.13 ± 1.4 days.

**Table 1:** Some features of the pediatric patients

Variable	N	%
**Gender**		
Female	38	30.6
Male	86	69.4
**Medical Diagnosis**		
Respiratory diseases	66	53.2
Gastrointestinal diseases	46	37.1
Urogenital diseases	10	8.1
Neurologic diseases	2	1.6

No growth in the initial culture was observed in 51.6% of the patients, 7.2% patients had a permanent flora, and 22.6% had *coagulase-negative staphylococcus* (CNS). The most common pathogenic microorganism in hand cultures was *Staphylococcus aureus* (11.3%) followed by *Streptococcus spp.* (3.2%). *Acinetobacter baumannii, S. epidermidis*, *Lactococcus garvieae, Proteus mirabilis*, and *Escherichia coli* were found in the hand flora of one patient (Table 2).

**Table 2:** Pathogenic microorganisms in the initial cultures and discharge cultures of the patients

	Initial Cultures		Discharge Cultures	
	
Species	N	%	N	%
No growth	64	51.6	52	41.9
Persistent Flora	9	7.2	-	-
CNS	28	22.6	24	19.4
MRCNS	-	-	19	15.3
*Acinetobacter baumannii*	1	0.8	11	8.9
*Staphylococcus aureus*	14	11.3	5	4.0
*Streptococcus spp*	4	3.2	-	-
*Staphylococcus Epidermidis*	1	0.8	-	-
*Lactococcus garvieae*	1	0.8	-	-
*Proteus mirabilis*	1	0.8	-	-
*Escherichia coli*	1	0.8	8	6.4
*Kocuria rhizophila*	-	-	1	0.8
*Kocuria kristinae*	-	-	1	0.8
*Candida*	-	-	1	0.8
*Pseudomonas spp*	-	-	1	0.8
*Enterococcus*	-	-	1	0.8

Total	124	100	124	100

Examination of the final discharge cultures showed reproduction in 72 of the patient cultures. CNS, *methicillin resistant-CNS* (MRCNS), *S. aureus, E. coli*, and *A. baumannii* were isolated from 19.4%, 15.3%, 4.0%, 6.4%, and 8.9% of the patients, respectively. Other pathogens detected in the cultures were *Kocuria kristinae* (0.8%), *Candida* (0.8%), *Pseudomonas spp. (*0.8%), and *Enterococcus* (0.8%) (Table 2). There is not a relation between children's disease and pathogens microorganisms.

## Discussion

This study was conducted to determine the microorganisms represented in the hand flora of pediatric patients. In present study, we observed growth of some bacteria which are resistant and may lead to long-term hospitalizations.

While no growth was seen in 51.6% of the initial cultures, a mixed flora and one of the most important pathogens of the hand flora, namely, CNS were detected in 7.2% and 22.6% of the initial cultures, respectively. No MRCNS was seen in the initial cultures of the patients; however, MRCNS was seen in 15.3% of the final discharge cultures of the patients. These results indicate that children may have been contaminated during hospitalization. Epidemiologic studies have shown that microorganisms are mostly transmitted via the hands. Inadequate hand hygiene is the most important cause of the hospital infections. Hanci et al. examined the hand cultures of the companions of the patients, and they detected MRCNS in 39% of the initial hand cultures of the patients, and this rate increased up to 40.5% after their visit (20). Akpınar et al. observed growth of MRCNS in 4.5% of the hand cultures of nursing students after clinical practice, but MRCNS was not observed in the initial culture (19). Half of the *coagulase-negative staphylococci* are methicillin-resistant and they are the third most common pathogens associated with hospital infections (19,20). *S. aureus* has been detected in both initial and final discharge cultures of the patients. It was found in the initial hand cultures of the 14 patients, and in the final discharge cultures of 5 patients (transmitted from the hospital). Price et al. showed that *S. aureus* was present in 58% of samples obtained from healthcare professionals at least once and in 8%–50% of environmental samples. In the same study, they detected transmission of *S. aureus* to 25 patients from healthcare professionals or environmental factors (2). In another study, a patient-to-patient transmission was determined in 7 adult Intensive Care Unit patients (21). Results of previous studies with adults (healthcare professionals, nurse students, companions of the patients) report contamination with pathogenic microorganisms related to the hospital. The results of our study are important in terms of being the first study showing contamination with pathogenic microorganisms in children hospitalized at pediatric units. The observation of *S. aureus* on the hand samples of pediatric patients in this study is an important finding. Pediatric patients should be considered as a special group. Because, they interact with other children and the environment rather than staying in their bed at the hospital. Children are always interacting with each other, medical devices (stethoscopes, thermometer, etc) and healthcare professionals in the unit that allows the microorganism to spread easily. *A. baumannii* was observed in the initial hand culture of one patient and in the final discharge cultures of 11 patients. *A. baumannii* is the most common species isolated from clinical specimens. They are a type of antibiotic-resistant bacteria that are usually isolated from soil and can survive in animals and on non-living things. They are rarely seen in epidemic infections; however, they are usually isolated in nosocomial infections (22,23). Most of the antibiotic-resistant strains of bacteria were isolated from the patients staying in the pediatric unit who especially require long-term treatment with broad-spectrum antibiotics. The presence of antibiotic-resistant bacteria in 11 children was a high and striking result.

Another notable finding in final discharge culture of the patients is *E. coli.* Bacterial growth of E. *coli.*, which is one of the most common causes of hospital infections and often results from inadequate hand hygiene, was seen in eight children (24,25). Doğukan et al. compared E. *coli* growth at faucet knobs and door handles (26). Although no study has been done in children's hospitals, gastroenteritis is the most important issue in daycare centers. Studies have shown that adequate hand hygiene provides a 30%–40% reduction in gastroenteritis (15–17). The most important cause of spread of infection among children is through door handles and toys (27). Children in daycare centers, similar to those in pediatric units have hand contact with each other and play with the same toys. These microorganisms may be transmitted through medical devices, door handles, toys or healthcare workers.

*Candida* growth was detected in a hand of child in the unit. *Candida* species can be found in clinics not only in invasive infection sites but also in sinks (28). A study on hand hygiene of the relatives of the patients showed that *Candida spp* was found on hand samples of two individuals after their hospital visit (20). In the same study, it was reported that *Enterococcus* was detected on the hands of 5 individuals after the visit, and the same microorganism was also found in a child in our study. In our study, different pathogens were seen in the initial and final discharge cultures of one individual. The initial culture was with *S. epidermidis, L. garvieae, P. mirabilis*, and *K. rhizophila, K. kristinae* and growth of *Pseudomonas spp* were observed in the final discharge cultures. Although to a small extent, the growth of these pathogens on the hands of children should be monitored. Children usually put their hands to their mouths, and they usually touch their open wounds or invasive interference areas. And, touching anywhere without cleaning the hand will increase the spread like a chain ring.

In this study, it was detected that pediatric patients were under a significant risk because of reproduction of many pathogenic microorganisms in their hand flora during their stay in the hospital. It was determined that there was an increase in the type and proportion of resistant microorganisms especially in cultures taken immediately before discharge. The cleaning of the touched areas (door handles, toys, medical devices like stethoscopes) and the provision of hand hygiene are necessary to prevent the spread of microorganisms. Based on the findings of this study, the provision of hand hygiene as well as clinical hygiene will be effective to reduce hospital infections.

The role of the hand flora of hospitalized children in the development of nosocomial infections is significant. The findings of the present study have practical importance in clinical practice to determined common microorganisms in the hand flora of hospitalized children. In the light of the findings of this study, it is important to take some precautions and develop policies about hand hygiene of pediatric hospitalized patients. For this purpose, healthcare professionals can provide effective hand hygiene training to both children and their mothers in the unit. The training given to children and their families should also contain toys, all the materials they bring with them and the materials they use in the unit. It may be effective to inform the healthcare professionals about the measures to be taken for patients about hand hygiene and hospital infections. In addition, alcohol-based hand disinfectants should be present in patients' rooms.

The results of our study are important in terms of being the first study showing contamination with pathogenic microorganisms in children hospitalized at pediatric units. It is believed that this pioneering study will provide the basis for other studies by creating speculative knowledge. It is recommended that the contamination of children should be investigated longitudinally.

## References

[1] Pereira EBS, Jorge MT, Oliveira EJ, Júnior ALR, Santos LRL, Mendes-Rodrigues C (2017). Evaluation of the multimodal strategy for improvement of hand hygiene as proposed by the World Health Organization. J Nurs Care Qual.

[2] Price JR, Cole K, Bexley A (2017). Transmission of Staphylococcus aureus between health-care workers, the environment, and patients in an intensive care unit: a longitudinal cohort study based on whole-genome sequencing. Lancet Infect Dis.

[3] Kouni S, Kourlaba G, Mougkou K, Maroudi S, Chavela B (2014). Assessment of hand hygiene resources and practices at the 2 children's hospitals in Greece. Pediatr Infect Dis J.

[4] Scheithauer S, Batzer B, Dangel M, Passweg J, Widmer A (2017). Workload even affects hand hygiene in a highly trained and well-staffed setting: a prospective 365/7/24 observational study. J Hosp Infect.

[5] Andersen BM, Rasch M, Kvist J (2009). Floor cleaning: effect on bacteria and organic materials in hospital rooms. J Hosp Infect.

[6] Özçetin M, Ulaş Saz E, Karapınar B, Özen S, Aydemir Ş, Vardar F (2009). Pediatric nosocomial infections; incidence, risk factors. Journal Pediatr Infect.

[7] Gulmez D, Gur D (2012). Microorganisms isolated from blood cultures in Hacettepe University İhsan Doğramacı Children's Hospital from 2000 to 2011: evaluation of 12 years - original investigation. Journal Pediatr Infect.

[8] Cavalcante SS, Mota E, Silva LR, Teixeira LF, Cavalcante LB (2006). Risk factors for developing nosocomial infections among pediatric patients. Pediatr Infect Dis J.

[9] Lakshmi KS, Jayashree M, Singhi S, Ray P (2007). No study of nosocomial primary bloodstream infections in a pediatric intensive care unit. J Trop Pediatr.

[10] Abramczyk ML, CarvalhoI WB, Carvalho ES, Medeiros EAS (2003). Nosocomial infection in a pediatric intensive care unit in a developing countryle. Brazilian J Infect Dis.

[11] El-Nawawy AA, El-Fattah MMA, Metwally HAE-R, Barakat SSED, Hassan IAR (2005). One year study of bacterial and fungal nosocomial infections among patients in pediatric intensive care unit (PICU) in Alexandria. J Trop Pediatr.

[12] Raymond J, Aujard Y (2000). Nosocomial infections in pediatric patients a european, multicenter prospective study. Infect Control Hosp Epidemiol.

[13] Mühlemann K, Franzini C, Aebi C (2004). Prevalence of nosocomial infections in swiss children's hospitals. Infect Control Hosp Epidemiol.

[14] Buettcher M, Heininger U (2010). Prospective surveillance of nosocomial viral infections during and after hospitalization at a university children's hospital. Pediatr Infect Dis J.

[15] Zomer TP, Erasmus V, Looman CW (2015). A hand hygiene intervention to reduce infections in child daycare: a randomized controlled trial. Epidemiol Infect.

[16] Mbakaya B, Lee P, Lee R (2017). Hand hygiene intervention strategies to reduce diarrhoea and respiratory infections among schoolchildren in developing countries: a systematic review. Int J Environ Res Public Health.

[17] van Beeck AHE, Zomer TP, van Beeck EF, Richardus JH, Voeten HACM, Erasmus V (2016). Children's hand hygiene behaviour and available facilities: an observational study in Dutch day care centres. Eur J Public Health.

[18] Çelik M (2017). Pediatri. Yurdakök.

[19] Akpınar RB, Celebioglu A, Uslu H, Hamidullah Uyanık M (2009). An evaluation of the hand and nasal flora of Turkish nursing students after clinical practice. J Clin Nurs.

[20] Hanci H, Ayyildiz A, Çelebi D (2012). Hasta ziyaretleri için hastaneye gelen kişilerin ziyaret öncesi ve sonrası el floralarının karşılaştırılması. Atatürk Üniversitesi Veterinerlik Bilim Derg.

[21] Price JR, Golubchik T, Cole K (2014). Whole-Genome sequencing shows that patient-to-patient transmission rarely accounts for acquisition of Staphylococcus aureus in an intensive care unit. Clin Infect Dis.

[22] Seruga Music M, Hrenovic J, Goic-Barisic I, Hunjak B, Skoric D, Ivankovic T (2017). Emission of extensively-drug-resistant Acinetobacter baumannii from hospital settings to the natural environment. J Hosp Infect.

[23] Roca I, Espinal P, Vila-Farrés X, Vila J (2012). The Acinetobacter baumannii Oxymoron: commensal hospital dweller turned pan-drug-resistant menace. Front Microbiol.

[24] Yilmaz K, Ermertcan Ş, Taşli H, Kurutepe S, Demiray T (2017). Pediatrik ve Yetişkin hastalardan izole edilen E. coli izolatlarının antimikrobiyal duyarlılıklarının ve virülans faktörlerinin irdelenmesi. Türk Sağlık Bilim Derg.

[25] MK, Yaman G, Göktaş U (2011). Hastane enfeksiyon etkenlerinin ve direnç profillerinin belirlenmesi. Van Tıp Derg.

[26] Doğukan M, Yaztürk Ş, Dilek AR (2007). Hastane kapı kolu ve musluklarının patojen bakteriyel kontaminasyon yönünden değerlendirilmesi. Fırat Üniversitesi Sağlık Bil Derg.

[27] Aiello A, Coulborn R, Perez V, Larson E (2008). Effect of hand hygiene on infectious disease risk in the community setting: a meta-analysis. Am J Public Health.

[28] Jencson AL, Cadnum JL, Piedrahita C, Donskey CJ (2017). Hospital sinks are a potential nosocomial source of candida infections. Clinical Infectious Diseases.

